# Model-Based Mid-Level Regulation for Assist-As-Needed Hierarchical Control of Wearable Robots: A Computational Study of Human-Robot Adaptation

**DOI:** 10.3390/robotics11010020

**Published:** 2022-01-29

**Authors:** Ali Nasr, Arash Hashemi, John McPhee

**Affiliations:** Department of Systems Design Engineering, University of Waterloo, Waterloo, ON N2L 3G1, Canada; arash.hashemi@uwaterloo.ca (A.H.); mcphee@uwaterloo.ca (J.M.)

**Keywords:** wearable robots, exoskeletons, biomechatronic system, human–robot interaction

## Abstract

The closed-loop human–robot system requires developing an effective robotic controller that considers models of both the human and the robot, as well as human adaptation to the robot. This paper develops a mid-level controller providing assist-as-needed (AAN) policies in a hierarchical control setting using two novel methods: model-based and fuzzy logic rule. The goal of AAN is to provide the required extra torque because of the robot’s dynamics and external load compared to the human limb free movement. The human–robot adaptation is simulated using a nonlinear model predictive controller (NMPC) as the human central nervous system (CNS) for three conditions of initial (the initial session of wearing the robot, without any previous experience), short-term (the entire first session, e.g., 45 min), and long-term experiences. The results showed that the two methods (model-based and fuzzy logic) outperform the traditional proportional method in providing AAN by considering distinctive human and robot models. Additionally, the CNS actuator model has difficulty in the initial experience and activates both antagonist and agonist muscles to reduce movement oscillations. In the long-term experience, the simulation shows no oscillation when the CNS NMPC learns the robot model and modifies its weights to simulate realistic human behavior. We found that the desired strength of the robot should be increased gradually to ignore unexpected human–robot interactions (e.g., robot vibration, human spasticity). The proposed mid-level controllers can be used for wearable assistive devices, exoskeletons, and rehabilitation robots.

## 1. Introduction

The human subject wearing a robotic assistive device that interacts with the environment forms a closed-loop system with two separate controllers: the human central nervous system (CNS) and the robot control system [1]. These two control systems simultaneously modify the behavior of the closed-loop system with different approaches of robot’s hierarchical control [2,3] and human’s optimal control [4]. Desirable performance of wearable devices entails good coordination between these two controllers [5]. This can be accomplished by developing an effective robot controller that considers both the wearer and the robot in its design. Additionally, the robot controller should be appropriately modified as the human adapts to the device to account for human–robot coadaptation. For this purpose, researchers have either conducted human-in-the-loop studies or personalized computer simulations [1,6,7,8]. Since safety is a major consideration in biomechatronics research, this paper adopts the latter approach; the results of this research pave the way for future experimental implementations.

**Figure 1:**
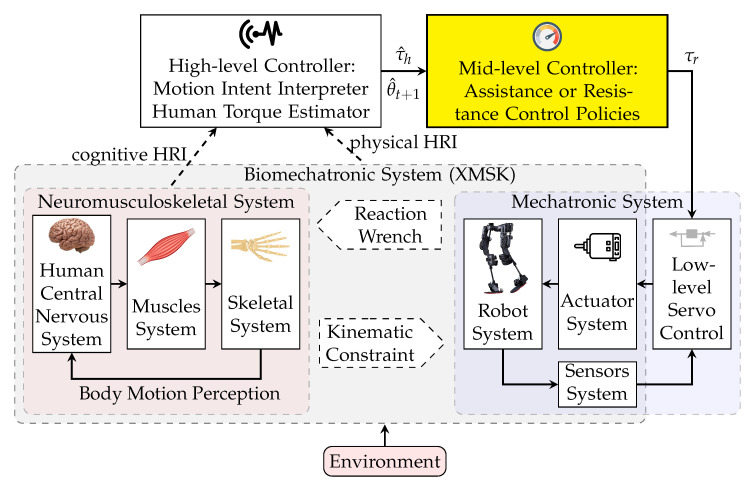
Schematic of human–robot–environment system with hierarchical strategy control. ${\hat{\tau }}_{h}$ is estimated human joint torque and ${\hat{\theta }}_{t+1}$ is predicted/desired joint angle computed by the high-level controller.

The state-of-the-art control of robotic rehabilitation, assistive, exoskeleton, and prosthesis systems utilizes a hierarchical design: high, mid, and low level controllers [3,9] (Figure 1). The high-level controller is responsible for interpreting the motion intent of the user (primarily for robotic prosthesis systems) or the exerted torque and force by the user (mainly for wearable robotic systems); the two approaches are categorized into cognitive human–robot interaction (cHRI) [10,11] and physical human–robot interaction (pHRI) [12,13]. The mid-level controller transforms the determined intended motion or wrench (force/torque) from the high-level controller into the desired motion or wrench trajectories for the low-level controller [14]. Finally, the low-level controller is responsible for following the desired states (from the mid-level controller) by considering the measured device states (from the robotic sensors) and directly commands the robotic actuators [3,9]. The current paper focuses on the mid-level controller.

Apart from the quality of the sensors, the accuracy of the interpreted intent/wrench, the actuator, and the low-level feedback control, the mid-level controller plays a vital role in users’ experience [15]. It governs the human–robot–environment interaction (HREI) [16]; the HREI depends on the robot’s mid-level cooperation control. Developing effective robotic systems requires the consideration of application targets (clinical or industrial), power augmenting purposes (resistive rehabilitation or power-assistive fixture), and the human interaction adaption level [15,16]. An inefficient control policy may limit the normal human range of motion or increase discomfort due to unnatural motion [17,18]. Executing natural motions from human intent is challenging, applying volitional natural human adjustment is difficult, and the overall applicability is limited [2]. Recently, the assist-as-needed (AAN) strategy, which operates based on enhancing motor activity but not replacing it, shows promising results [7,12,19,20,21].

So far, common strategies have been used for controlling wearable robots: finite state machines/prerecorded motion [22,23], master–slave [16], proportional [24,25], fuzzy mapping [26,27,28], and combination control strategies [14,29]. The preprogrammed method is mainly used for cyclic movements [23] (e.g., walking, running, and stair climbing) or classified movements (e.g., hand/finger posture), and the user only activates the strategy at the start. The controller does not utilize feedback during the maneuver, which is non-volitional. Although the master–slave control uses human motions and simultaneously commands the same motions, the robot wearer feels an uncomfortable contact force if there is a human–robot size inconsistency [16]. A proportional controller was established to produce a proportional control signal from EMG signals [10,11,24,25,30]. However, the EMG–torque relation is not proportional, and, hence, using this approach makes an uncomfortable motion for the wearer. The decision-based fuzzy controller system was developed to avoid the complex dynamic modeling system [26,27,28]. However, generalizing this method is difficult due to different subject biomechanical signals. Researchers have used a hybrid assistive control since signal-based control strategies are inefficient for different motions [31]. Depending on the motion requirements, the controller was segmented into various control phases [31].

Overall, the inefficiency, application limitation, uncomfortable movement, and usage anxiety challenges are still important issues to resolve [15,17]. They result from the following: ignoring the limb dynamics or assuming constant limb dynamics, neglecting the robot stiffness and inertia, adapting to an overly simplified strategy that does not capture the mathematical relationships between variables, and struggling with tuning/adjusting the controller for participants. The system dynamics play an essential role since the applied torque to robots, external wrench (from disturbance or manipulation object), and the reaction wrench on the user lead to the motion according to the system dynamics [18]. The sources of human–robot–environment system dynamics consist of inertial properties of human segments and robot links (proportional to the joint acceleration, according to Newton’s law), centripetal force (proportional to the joint velocity squared and toward the center of rotation), Coriolis effect (interaction of two rotating links/limb), gravity wrench (caused by gravity), and external wrench (caused by friction, environment, or heavy object handling).

For example, in the presence of an external load, the amount of necessary activation torque increases compared to the free human limb movement. The robot’s dynamics (stiffness and inertia) and the environment/external load result in extra necessary torque. The AAN mid-level control of wearable robots should be designed to provide this extra torque [19,21]. As a result, the primary goal of this project is to create a model for a mid-level controller that includes two distinct parts: the robot and the environment. This method is similar to a combination of torque and gravity compensation control approaches [14,32].

Previous research has converted the human–robot–environment interaction system to an overly simplified rule. Consequently, adjustments are necessary for any changes in the dynamics of limb, robot, and environment [2,24,27,31]. To overcome these challenges, or at least mitigate the inefficiencies and limitations, considering the human–robot–environment dynamic interaction is a viable solution.

The human CNS, involving the brain and the spinal cord, controls the human body’s motion [4,33,34]. It concurrently manages the kinetics and the kinematics, despite uncertain/unknown trajectories and complicated muscle dynamics [4,35]. According to experimental studies of human movement, it is hypothesized that the CNS coordinates the body movement in an optimal manner [36,37]. Multiple research has postulated that the CNS obtains the optimal inputs by minimizing a cost function that can include jerk [38], torque [39], muscle activation [40], metabolic energy [41], and muscle fatigue [42] terms. However, in most mid-level controller assessment studies, the central neural system is modeled with a Proportional-Integral-Derivative (PID) controller [43,44]. The wearer can adapt to the robot dynamics and the controller in the long term, yet the adaptation level is low in the initial and short-term experiences. We aim to test a mid-level controller by modeling the CNS as an optimal controller [4] for the initial, short-term, and long-term experiences.

The distinguishing novelties and contributions of this work are:

1. Consideration of the distinctive effect of the human’s and robot’s dynamic models, as well as the wrench of the environment;
2. Optimization, evaluation, and comparison of a proportional, a novel model-based, and a novel fuzzy-logic mid-level controller for assist-as-needed control of a wearable robot during two free motion and lifting tasks;
3. Assessment of the three mid-level controllers for three phases of (A) initial, (B) short-term, (C) long-term experiences of wearing a powered robot.

In this paper, the mid-level controller within a hierarchical strategy in assistance-resistance control is first introduced with three methods of proportional, model-based, and fuzzy-logic rule. Second, the CNS controller is described for studying the adaptation. Third, the results and the evaluation of the controllers on the system of human–robot–environment are presented. Finally, the effectiveness of controllers for AAN goals and initial, short, and long-term experiences is discussed.

## 2. Mid-Level Controller

The input variables to the mid-level controller are from the high-level controller, such as estimated human joint torque ${\hat{\tau }}_{h}$ and the system kinematic feedback measured by sensors, such as predicted/desired joint angle ${\hat{q}}_{d}$, velocity ${\hat{p}}_{d}$, and acceleration $\frac{d{\hat{p}}_{d}}{dt}$, feedback/measured joint angle $q_{f}$, velocity $p_{f}$, and acceleration $\frac{dp_{f}}{dt}$. The strength and direction of the applied wrench are defined in the mid-level controller. The user can define the strength of assistance or resistance Ω. The output of this controller is the desired robot torque $\tau_{r}$, which is commanded to the low-level controller.

In this research, two new model-based and fuzzy-logic-based mid-level controllers are introduced, considering the distinctive effect of the human and the robot’s dynamic models. The common proportional rule is also applied for comparison with the above two approaches. For a fair comparison, the gain of all controllers is optimized.

### 2.1. Proportional Rule

**Figure 2:**
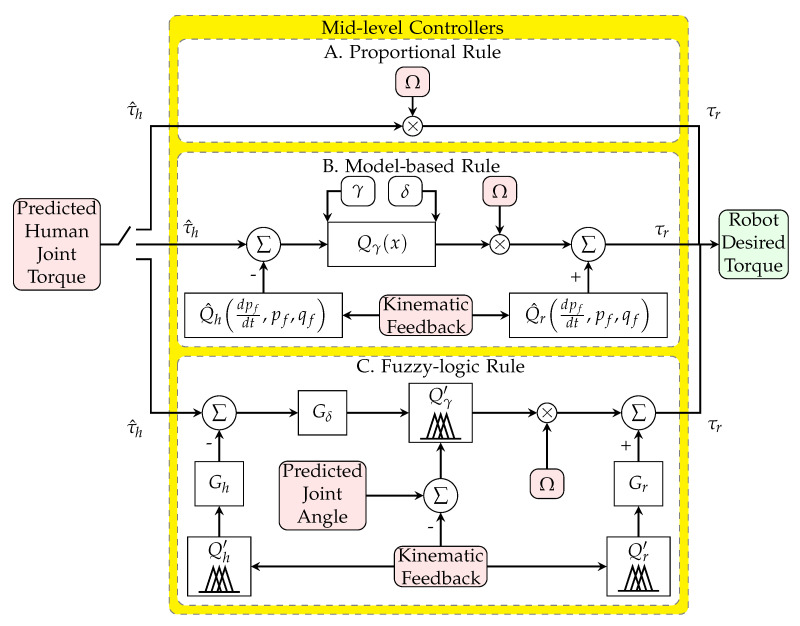
Schematic of proportional rule (**A**), model-based rule (**B**), and fuzzy-logic rule (**C**) as mid-level controller.

Researchers have commonly used the proportional gain method [2,24,25] (Figure 2A). The desired robot torque $\tau_{r}$ is calculated as a proportion of the estimated human joint torque ${\hat{\tau }}_{h}$ (Equation (1)). This conventional controller does not consider distinctive human, robot, and environment system models. Directly using the human torque as the command of the robot torque causes subject discomfort in exoskeletons, as described in Section 1.

(1) $$\tau_{r}=\Omega {\hat{\tau }}_{h}$$

### 2.2. Model-Based Rule

This novel approach uses the identified limb model and the robot model to find the net torque of the human joint without the shared portion for the limb and the robot fixture; this approach resolves the previously mentioned issues by considering the human and robot dynamics. The net torque may multiply to the desired strength variable to further increase or decrease assistance or resistance (Figure 2B). The detailed model has been presented in Equations (2)–(4).

(2) $$\tau_{r}=\Omega Q_{\gamma }\left(\tau_{\delta }\right)+{\hat{Q}}_{r}\left(\frac{dp_{f}}{dt},p_{f},q_{f}\right)$$

(3) $$Q_{\gamma }\left(\tau_{\delta }\right)=\tau_{\delta }\left(\frac{1}{1+e^{-\frac{4}{\gamma -\delta }\left(\tau_{\delta }-\frac{\gamma +\delta }{2}\right)}}+\frac{1}{1+e^{\frac{4}{\gamma -\delta }\left(\tau_{\delta }+\frac{\gamma +\delta }{2}\right)}}\right)$$

(4) $$\tau_{\delta }={\hat{\tau }}_{h}-{\hat{Q}}_{h}\left(\frac{dp_{f}}{dt},p_{f},q_{f}\right)$$

where ${\hat{Q}}_{r}$ and ${\hat{Q}}_{h}$ are the calculated torque from the wearable robot model and the human limb model (without the external load impact), respectively. We used a 1-DoF limb model with an elevation joint variable for ${\hat{Q}}_{r}$ and ${\hat{Q}}_{h}$ for simplicity. The 1-DoF limb model is selected so that our simulations are better aligned with our exoskeleton physical setup, which only has a shoulder joint kinematic sensor and cannot measure the elbow joint angle. $Q_{\gamma }$ is the model-based threshold torque function that eliminates the controller chattering effect. It is defined by a hyperbolic relation in Equation (3). γ and δ denote the threshold value and the dead zone, respectively. The effect of the dead zone becomes more significant as the accuracy decreases for the ${\hat{\tau }}_{h}$ and ${\hat{Q}}_{h}$ estimates. The threshold γ should not be more than the maximum torque of the robot actuator. Ω is the desired strength variable, which is negative for resistive and positive for assistive control. $\tau_{\delta }$ introduced in Equation (4) is the difference between the estimated human joint torque and the torque of the human limb model.

### 2.3. Fuzzy-Logic Rule

**Figure 3:**
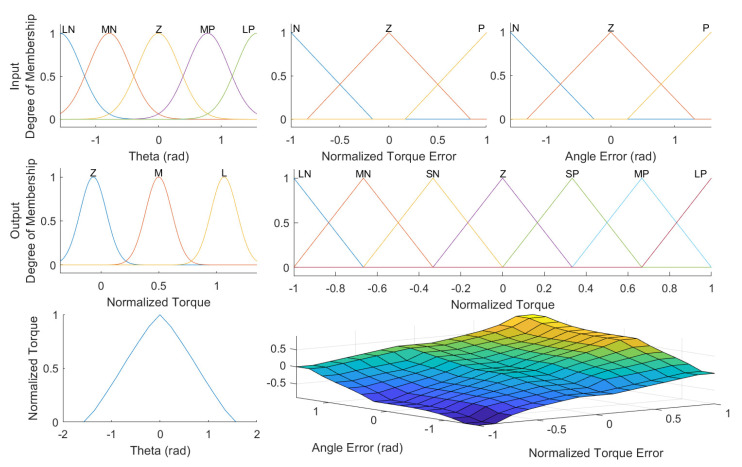
The membership function and degree of membership for the two models with three inputs (**top**) and two outputs (**middle**). The surface function plots of the outputs of the models for all input variables (**bottom**).

The model-based control method (Section 2.2) can be implemented by fuzzy logic rules that regulate the actuator. The detailed novel model is presented in Equation (5) and is shown in Figure 2C.

(5) $$\tau_{r}=\Omega {\overset{´}{Q}}_{\gamma }\left({\overset{´}{\tau }}_{\delta },q_{d}-q_{f}\right)+{G_{r}\overset{´}{Q}}_{r}$$

(6) $${\overset{´}{\tau }}_{\delta }=G_{\delta }\left[{\hat{\tau }}_{h}-{G_{h}\overset{´}{Q}}_{h}\right]$$

where Ω, ${\overset{´}{Q}}_{\gamma }$, and ${\overset{´}{Q}}_{r}$ are the strength, threshold, and robot gravity function, respectively. The three functions are defined in Table 1, using the fuzzy-logic condition-statement rules. The rules can be more complex and use more feedback, such as velocity $p_{f}$ or acceleration $\frac{dp_{f}}{dt}$. ${\overset{´}{\tau }}_{\delta }$ is the difference between the estimated human joint torque and the torque of the human limb model, which is defined in Equation (6). $G_{r}$, $G_{h}$, and $G_{\delta }$ are the gains of the robot fuzzy-logic model, human fuzzy-logic model, and environment fuzzy-logic model, respectively; Ω is the desired strength variable. The degree of membership and membership function for the two models (the three inputs and two output), as well as the surface plot of the input–output functions are shown in Figure 3.

## 3. Control System Evaluation

**Figure 4:**
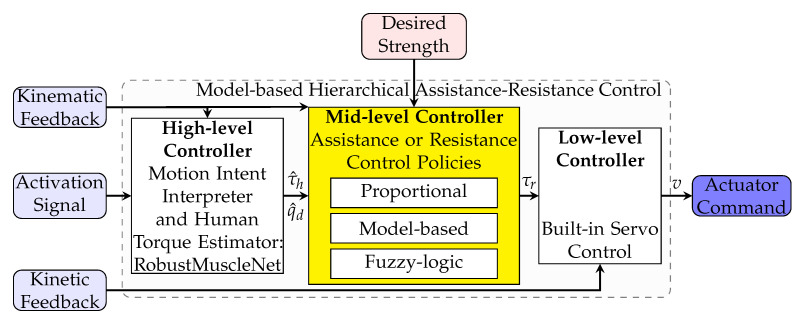
Schematic of the hierarchical control. The external input signals are kinematic feedback, activation or sEMG signal, and kinetic feedback. The mid-level controller coordinates the desired assistive torque based on the desired strength.

**Figure 5:**
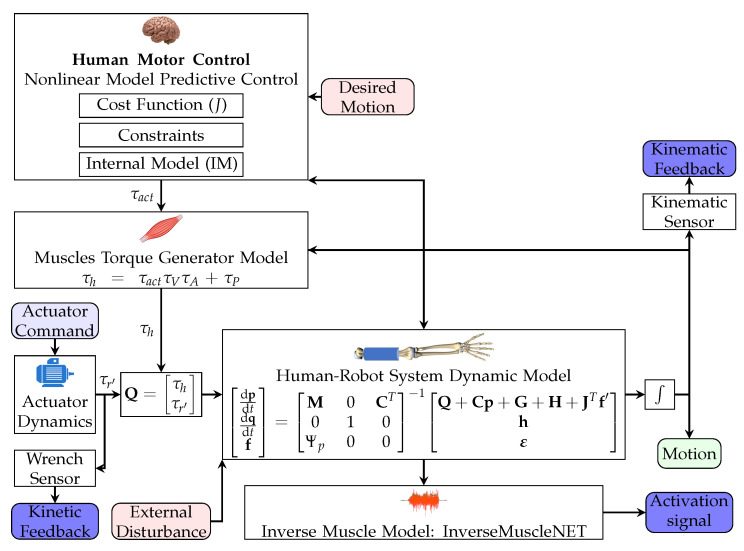
Schematic of the human–robot multibody system. The CNS is represented by the NMPC controller. The MTG model relates the torque to joint angle and angular speed. The InverseMuscleNET block estimates the sEMG signals.

To evaluate the robot control, it is essential to test it on a virtual system before experimental testing with humans. The virtual system should consist of a human, a robot, and an environment model, which should simulate systems and produce kinematic feedback, kinetic feedback, and activation signals. The inputs to this system are the robot’s actuator command and the desired motion (shown in Figure 4). The schematic of the multibody system of the human and the wearable robot is depicted in Figure 5.

The dynamic system model consists of the human skeletal model and the robot dynamics in Equation (7).

(7) $$\left[\begin{array}{ccc} M & 0 & C^{T}\\ 0 & 1 & 0\\ \Psi_{p} & 0 & 0 \end{array}\right]\left[\begin{array}{c} \frac{dp}{dt}\\ \frac{dq}{dt}\\ f \end{array}\right]=\left[\begin{array}{c} Q+\mathrm{Cp}+G+H+J^{T}\overset{´}{f}\\ h\\ \varepsilon \end{array}\right]$$

where M, C, and Ψ are the mass matrix (n×n), constraint reactions coefficient matrix (m×n), and the Jacobian matrix of the velocity constraint equations concerning the generalized velocity p. Q, C, G, and H are the applied wrench, relevant matrix to Coriolis effect and centrifugal force, gravity vector, and the vector of further joint torques from the inherent stiffness and friction, respectively. J and $\overset{´}{f}$ are the Jacobian matrix relevant to human–environment system force/torque locations, and the vector of the external forces and torques of the environment. h, ε, and f are the right-hand side of transformation between the derivative of coordinates and generalized speeds, the error, and the reaction wrench that enforces the kinematic constraint equations.

The activation signal, which is estimated via InverseMuscleNET, maps the human activation torque, joint torque, joint angle, joint velocity, and joint acceleration to the activation signals or EMG signals [45].

The performance of muscles and joint constraints have been modeled using muscle torque generators (MTGs) [46]. An MTG model allows simulation of the muscle model components without modeling individual muscle forces by, for example, the Hill-type muscle model [47]. Explicitly, the components of muscle modeling consist of the joint angle relation (passive behavior of muscle) and the joint velocity relation (dynamic behavior of muscle) [46,48]. MTG model is exhibited in:

(8) $$\tau_{h}=\tau_{act}\tau_{a}\tau_{v}+\tau_{p}$$

where $\tau_{a}$, $\tau_{v}$, and $\tau_{p}$ are the position-scaling function, the velocity-scaling function, and the passive torque function, respectively. $\tau_{act}$ and $\tau_{h}$ are the input activation torque and the total human joint torque as output, respectively.

As mentioned earlier, the CNS coordinates human arm motion by complex commands, which are the mixture of (I) the motion prediction and (II) the corrective command [4,33,34]. (I) the motion prediction or feed-forward control is calculated from an internal model or representation of the complex system. (II) the corrective command or feedback control is computed from the sensory organs to correct any errors due to model uncertainty, external disturbance, or unknown environment. NMPC can achieve this multiple-purpose control complexity (feed forward and feedback control) with an infinite or finite (as in our case) horizon formulation. The NMPC uses an internal model (IM) to represent the human dynamics for predicting the optimal motion (feed forward) and sensory information for correcting the prediction errors (feedback) [4]. It utilizes a cost function to evaluate the optimal motion. This cost function is a combination of joint angle and velocity errors, as well as the torque and the torque derivative:

(9) $$J=\int_{0}^{t_{f}}\omega_{1}^{T}{\left(q-q_{d}\right)}^{2}+\omega_{2}^{T}{\left(p-p_{d}\right)}^{2}+\omega_{3}^{T}{\left(\tau_{act}\right)}^{2}+\omega_{4}^{T}{\left({\dot{\tau }}_{act}\right)}^{2}$$

(10) $$Subject\;to:\;\begin{array}{c} \begin{array}{c} q_{min}\leq q\leq q_{max}\\ p_{min}\leq p\leq p_{max} \end{array}\\ \begin{array}{c} \tau_{min}\leq \tau_{act}\leq \tau_{max}\\ {\dot{\tau }}_{min}\leq {\dot{\tau }}_{act}\leq {\dot{\tau }}_{max} \end{array} \end{array}$$

The CNS learns and identifies the IM in the long term [49,50], and the muscles can adapt in the short-term [50,51,52]. Clearly, after the initial use of a wearable robot, the CNS only has information about the IM of the human and the old weights for the limb, and has no model for interacting with the wearable robot. In short experiences, the human can adjust and tune the weights for the new condition, subject to the wearable robot’s assistive or resistive torque [53]. We use the human limb model as the IM for the initial and short experiences. The IM incorporates the robot dynamics in the long-term experience. In addition, the weights ($\omega_{i}$) are tuned for free limb motion only and are used for the initial experience. The weights are adjusted for the short-term and long-term experience.

The weights in the cost function (Equation (9)) are manually adjusted for three conditions of (I) initial, (II) short-term, and (III) long-term experience (Table 2). The initial experience shows the human–robot interaction but has the same weights as the human-only mode. The short-term experience is when the IM is not updated, and the weights are adjusted for the optimal motion. The long-term experience is when the robot dynamics and mid-level controller are incorporated into the IM, i.e., the human has adapted to the robot.

## 4. Case Study

We have used a two degree-of-freedom (DOF) limb model with segments for the upper and the lower arm as the human skeletal model [54]. In addition, as mentioned in Section 2.2, the exoskeleton is providing assistance and measuring the joint angle only for the human shoulder joint. The human elbow joint has no assistance and no sensor for the robot’s controller; this is to make the case study model similar to our physical setup. This human skeletal model has been equipped with a shoulder exoskeleton for assisting the elevation angle. The position-scaling, velocity-scaling, and passive functions, employed from [48,55], use dynamometry measurements for the shoulder and elbow joint.

The IM of the NMPC only has the limb model (without the exoskeleton model). The controller constraints are biomechanically-inspired limits, such as minimum and maximum range of motion, joint velocity, joint torque [48,55], and an assumed joint torque rate of 5 (Nm/s).

**Figure 6:**
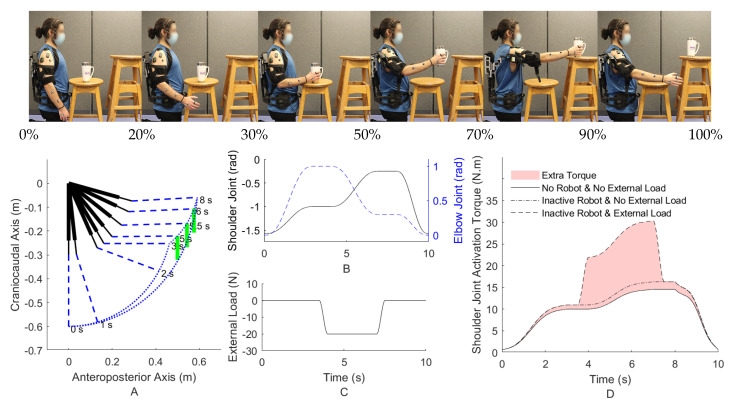
The motion capture system markers (Vicon Motion Systems Ltd, UK) and wireless EMG sensors (Delsys Inc, MA, USA) are attached to the subject who is wearing an inactive EVO exoskeleton (Ekso Bionics Holdings Inc., CA, USA) (**top**); The sagittal pick and place task with active shoulder and elbow joint (**A**). The desired trajectory of the shoulder and elbow joint angles (**B**). The external vertical load of the object at the palm of the hand (**C**). The required activation torque of the shoulder joint with and without an inactive robot using inverse dynamic simulation (**D**).

An instance of the task motion and the desired trajectory (consisting of two different tasks of free motion and lifting) for an object pick-and-place in the sagittal plane subject to an external load is shown at the top of Figure 6 and in Figure 6A. Other reference trajectory planning methods, such as regression analysis using a Kinect and point cloud, can also be used [56]. In addition, the optimal desired trajectory can be obtained by defining the start and end points and then solving an optimization problem comprising cost function terms from the robot (e.g., minimize robot input and jerk) and the subject (e.g., minimize joint acceleration or muscle activation) [57].

In addition to the task-space path of the palm of the hand in Figure 6A, the shoulder and elbow joint angles are obtained using the inverse kinematic geometric calculation and shown in Figure 6B. The vertical force that is transferred to the palm of the hand due to gravity and the object weight is shown in Figure 6C. As depicted, the force is −20.0 N starting from the grasping time and goes back to zero at release.

Different activation torques of the shoulder joint in different conditions are shown using the inverse dynamic simulation of the skeletal-MTG model in Figure 6D. The different conditions consist of (I) not wearing an assistive shoulder exoskeleton and no presence of the external load (motion-only), (II) wearing an inactive exoskeleton with no presence of the external load, and (III) using an inactive exoskeleton subject to the external load of an object. Evidently, wearing an inactive robot requires more joint torque since the robot joint has stiffness and the robot link has inertia (the lower highlighted area in Figure 6D). Additionally, the amount of the required activation torque increases in the presence of the external load. Thus, the sources of extra required torque are the dynamics of the robot (stiffness and inertia) and the environment/external load. The goal of the AAN mid-level control of wearable robots is to provide this extra required torque. Consequently, the main reason for this work is to provide a model with two distinct parts of the robot and the environment.

The criterion for assessing the proficiency of each mid-level controller is how accurately they can provide the required extra torque with the presence of the robot and environment load. If the robot provides an excessive assistive torque, the human joint will provide less torque than the no-robot condition (when the skeletal dynamics is performing the motion without the robot and the external load). This lower than normal human joint torque results in discomfort; for example, it suggests to the CNS that the inertia of the human limb has decreased from the usual amount [17].

For a fair comparison of the mid-level controllers, each mid-level controller’s gains and parameters should be optimized. The cost function of this optimization is decreasing the highlighted area in Figure 6D, or simply the difference between the extra required torque in the presence of the external load and inactive robot and the condition of not wearing the inactive robot and no external load. The particle swarm optimization method has been used for this nonconvex optimization with a swarm size of 200 for each variable. The NMPC adjusts for long-term experience in this optimization.

## 5. Results and Discussion

### 5.1. Full Strength (Fist Scenario)

**Figure 7:**
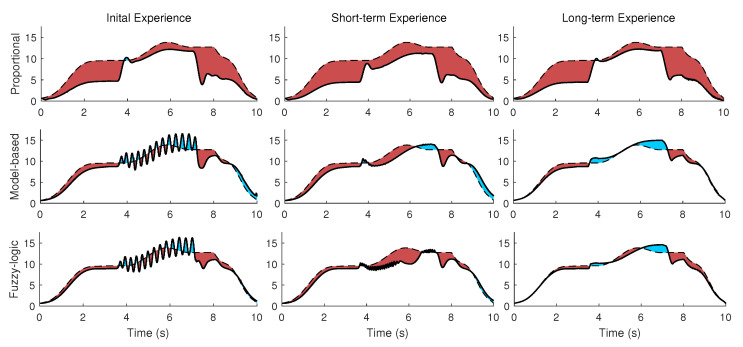
The 1st scenario (full strength) plot of the shoulder actuation torques (solid line), the desired shoulder actuation torque (dash line), the extra assistance (red area), and insufficient torque (blue area). Shown are the three mid-level optimized controllers of the proportional (**top-row**), model-based (**middle-row**), fuzzy-logic rule (**bottom-rule**) for three different conditions of initial (**left column**), short-term (**middle column**), long-term experience (**right column**) of wearing a powered robot.

The three mid-level optimized controllers of (A) proportional, (B) model-based, (C) fuzzy-logic rule for three different conditions of (I) initial, (II) short-term, (III) long-term experience of wearing a powered robot have been simulated (9 simulations in total). The activation torques of the shoulder joint using an inverse dynamic simulation for three conditions (1. No robot and external load, 2. Inactive robot and no external load, and 3. Inactive robot with external load) are shown in Figure 7. The goal is to decrease both the red (extra assistance) and blue (insufficient torque) highlighted areas in Figure 7.

**Figure 8:**
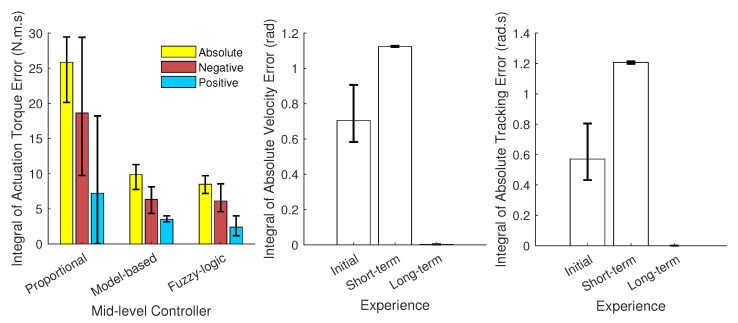
The integral of absolute, negative, and positive actuation error for proportional, model-based, and fuzzy-logic controller (**left**); The integral of absolute velocity error for three CNS conditions of initial, short-term, and long-term experience (**middle**); The integral of absolute tracking error for three CNS conditions of initial, short-term, and long-term experience (**right**).

As shown in Figure 6 and Figure 7, the desired actuation torque is equal to the condition that the human does not wear the robot and moves without experiencing the external load. Both model-based and fuzzy-logic mid-level controllers have a small error with the desired actuation torque (Figure 8). Particularly, the actuation torques of the proportional, model-based and fuzzy-logic mid-level controllers have 0.817, 0.311, and 0.268 Nm root-mean-square error, respectively. They consider the distinctive effects of the human’s dynamic model, robot’s dynamic model, and the required wrench of the environment. The proportional controller only has a specific gain to the human identified torque and provides more than necessary torque. This unnecessary torque can be seen in Figure 7; specifically, the human shoulder actuation torque decreases too much from the normal condition (highlighted in red). This extra torque means that humans tolerate less torque than expected in the same motion without the external load. After using the proportional controller for the long term and adapting to this controller, the user feels different dynamics after taking off the robot. Thus, it takes a while for the user to adapt to the normal condition. On the contrary, if the robot only provides part of the required torque, the human should provide the remaining amount, which leads to fatigue in muscles and decreases the efficiency of the wearable assistive robot.

The errors in the model-based rule result from the 1-DoF ${\hat{Q}}_{h}\left(\frac{dp_{f}}{dt},p_{f},q_{f}\right)$ model in Equation (4) without having the velocity and acceleration feedback, instead of a 2-DoF human arm, to mimic the practical complexity of robotic sensing. Specifically, as mentioned in Section 2.2, since the robot assists only the shoulder and has a built-in shoulder joint sensor, we only use the shoulder joint angle for mid-level control input. Then, ${\hat{Q}}_{h}\left(\frac{dp_{f}}{dt},p_{f},q_{f}\right)$ is considered as a 1-DoF limb with the shoulder joint kinematic data as an input variable. Practically, the 2-DoF human arm cannot be simplified to a 1-DoF system without error.

The robot’s mid-level controllers should not have a negative impact on the CNS and IM. Providing too much assistive torque suggests to the CNS that the upper-limb dynamics have been changed, for example, less inertia or less stiffness at the joint [17]. This problem can cause discomfort in industry workers every time they wear the exoskeleton [18]. Additionally, this issue can cause changes in the human sensation of inertia/stiffness even after the workers remove the exoskeleton.

The model-based and fuzzy-logic controllers have oscillation or vibration (velocity error) since they are based on the joint angle and velocity (Figure 7 and Figure 8). The proportional controller is based on the identified torque and has no chattering. The initial experience is evaluated with the NMPC-controlled CNS, and the weights are manually tuned for a no-robot condition. As shown in Figure 7, assisting when the human has not learned the interaction leads to vibration in motion (here, oscillation occurs about the shoulder joint frontal axis). The short-term experience has been simulated with the same weights for the NMPC except for the $\omega_{4}$ (weight of the torque derivative), which has been doubled (Table 2). Doubling the weight of the torque derivative means the human has activated both agonist and antagonist’s muscles to have a robust motion (with increasing muscle stiffness) [58,59,60]. This condition results in muscle fatigue since both kinds of muscles are activated [61]. In addition, when the weight of torque derivative increases, the impact of the other weight decreases in the cost function, so the tracking error increases in the short-term experience (Figure 8) [62,63,64].

The weights in Equation (9) have been manually adjusted, and the IM (internal model) knows the robot assistive torque in the long-term experience (Figure 7). Consequently, the vibration does not occur in the long-term experience. This awareness or adaption requires using the robot and motor learning for the long-term experience. One solution to mitigate the vibration in the initial experience is using a lower desired strength Ω for the robot controller in the first moments of using the robot and then increasing it after a while. This approach, i.e., changing the strength from less (initial experience) to high amount (long-term), has been experimentally evaluated in the literature [2,65,66], and the results have reported that the method of increasing the strength over time is preferred for human adaptation to robots. Many wearable biomechatronic device users have preferred to initiate with lower controller strength [65,66]. Then, whenever users adapt to the wearable device or become confident with practice after a while, they gradually tend to increase the controller gains/strength.

### 5.2. Variable Strength (Second Scenario)

**Figure 9:**
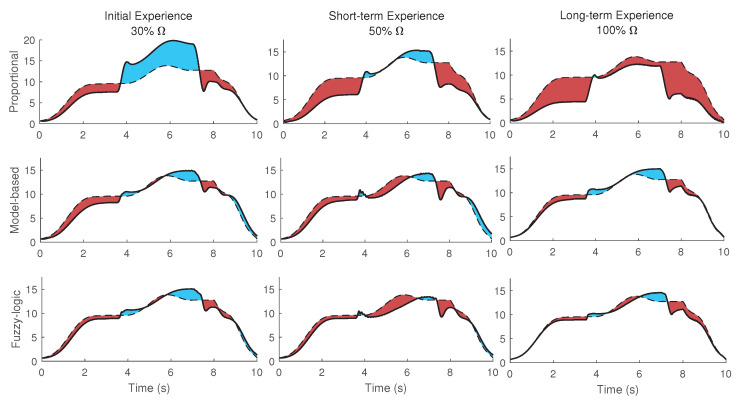
The 2nd scenario (variable strength) plot of the shoulder actuation torques (solid line), the desired shoulder actuation torque (dash line), the extra assistance (red area), and insufficient torque (blue area). Shown are the three different conditions of initial (**left column**) with 30% strength, short-term (**middle column**) with 50% strength, long-term experience (**right column**) with 100% strength.

To decrease the vibration about the frontal axis of the shoulder in the initial experience (shown on the left section of Figure 7), the simulation of controllers with 30% strength for the initial experience and 50% strength for the short-term experience (2nd scenario) is shown in Figure 9 (unlike the previous scenarios in which the strength was 100%). The long-term experience uses the full strength optimized for the long-term. As shown in Figure 9, unlike the initial experience section of Figure 7, the vibration does not happen. Since the vibration in the initial experience still has a minor impact, $\omega_{4}$ (weight of the torque derivative) for the short-term experience is 1.5 times of the weight for the initial experience. This value was used to increase 100% (from initial to short-term experience) for the condition in which full strength has been used in Figure 7. Thus, muscles are relaxed, and increasing muscle robustness is not required as in the previous scenario. Note that the assistance with 30% and 50% strength does not satisfy the goal of decreasing the area in Figure 6D. For example, in the initial experience for the proportional mid-level controller with 30% strength, the AAN is not satisfied with the mentioned criteria (the highlighted area from the desired value) 80% worse than a long-term experience for this mid-level controller. The simulated controller/model is required to provide most of the torque for handling the object for the period of 4–7 s.

### 5.3. Comparison

**Figure 10:**
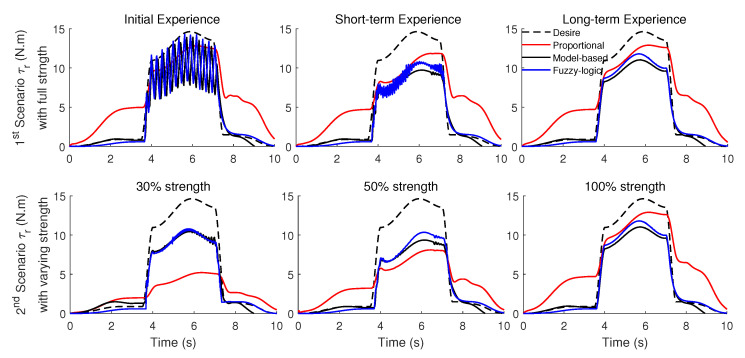
The desired robot torque is computed by the simulated inverse dynamic model (dashed), as well as the robot torque for proportional (red), model-based (black), and fuzzy-logic (blue) mid-level controllers. The first scenario with full strength and optimized weights for the short-term experience shows at top-row, and the second scenario with varying strength of 30% for initial experience at the left side, 50% for short-term experience at the middle side, and 100% for the long-term experience at the right side.

The robot torque for both scenarios (full strength and varying strength) is shown in Figure 10. The desired robot torque is computed with the inverse dynamic model simulation that is highlighted in Figure 6D. The proportional mid-level controller provides too much assistance for the free motion phase (0–3.5 and 7.5–10 s). In the case of 30% and 50% strengths, the proportional mid-level controller’s efficiency (following the desired torque) is much lower than the desired robot torque. Although the strength is variable in the second scenario, the model-based and fuzzy-logic mid-level controllers’ efficiencies do not decrease as much as the proportional mid-level controller for the lifting phase (3.5–7.5 s).

Another approach to reduce vibrations is relaxing the threshold value (γ) and the dead zone (δ) for the initial and short-term experiences. The result of increasing γ and δ lead to a similar simulation to Figure 9, which makes a new advantage for this model-based mid-level control. Instead of decreasing the strength of the mid-level controller, the threshold and the dead zone can be increased, but the strength can remain the same. A third possible solution to reducing the vibrations may be using an adaptive controller [43] or using a model predictive controller (MPC) with dual purposes of decreasing the joint error and the human-provided torque for the motion.

The model-based and fuzzy-logic controllers are based on the joint angle and velocity. Both can successfully provide the assistive torque required due to external load and the robot’s dynamic. In terms of comparison, the model-based controller with the capability of tuning the threshold value and the dead zone can slightly outperform the fuzzy-logic controller. In the case of a nonlinear limb model or higher-speed motion, the model-based controller can have better results than the fuzzy-logic controller, which uses linear regulation. In comparison to [26,27,28], our fuzzy-logic controller in Equations (5) and (6) is straightforward and simpler to implement.

### 5.4. Outcome and Future Work

In this study, the novel model-based and novel fuzzy-logic controllers are compared to proportional model-based controllers. The idea behind the proposed mid-level controllers is to consider both the human and the robot models. Taking into consideration models of the human and the robot, as well as using a nonlinear controller (e.g., model-based, fuzzy-logic, impedance control [67,68,69], haptic/admittance control [30,70], and adaptive control [43,71]) improves the efficiency of AAN control of wearable robots during two different tasks of free motion and lifting. The nonlinear controllers should be prioritized over proportional controllers for controlling the nonlinear human limb model.

So far, one major challenge of real-time control is the controller speed and computation delay [69,72]. The proposed controllers capture the interaction between the robot and the subject, and are also computationally tractable and applicable for real-time control.

Overall, this work’s distinguishing novelties and contributions are categorized into three points. (I) The effect of the human’s and robot’s dynamic model and the required wrench of the environment are considered in the design of the mid-level controller, for example, in Equations (2)–(4). (II) the strengths, variables, weights, and gains of an AAN wearable robot are optimized for two tasks of free motion and lifting (decreasing the area in Figure 6D). They are evaluated for three proportional, model-based, and fuzzy-logic mid-level controllers. (III) the three mid-level controllers for three phases of (A) initial, (B) short-term, (C) long-term experiences of wearing a powered robot are compared and assessed. Two solutions to the vibration of controllers and muscle fatigue are evaluated: decreasing the mid-level controller strength and relaxing the threshold value and the dead zone of the model-based mid-level controller.

To use this controller, an interface should be designed to capture the subject data and the desired strength variable Ω. Subject anthropometric data, including limb measurement and inertia parameters, will be incorporated in the $Q_{h}$ term in Equations (4) and (6), in a future implementation. In addition, there should be a calibration scenario to adjust the high-level controller model and parameters for each subject. On-board joint angle sensor and EMG sensor, as well as vision-based sensors, will be used to estimate the human joint torque or intention [2,73,74].

In a practical experiment, the noise and drift of the sensors may degrade the performance of the model-based and fuzzy logic controllers. Consequently, it is essential to make the control robust to any undesired motion [22]. In the future, the nonlinear mid-level controllers should be evaluated and enriched with a robust and safe term for human usage goals [18]. For example, to prevent rapid assistive torque changes due to noisy feedback signals, a low-pass filter or limiter can increase the robot’s safety by preventing rapid assistive torque changes due to noisy feedback signals.

In rehabilitation therapy applications, the desired strength variable Ω should gradually change from a small negative value to a bigger one. In addition, visual tracking has recently been utilized to evaluate the patient’s motor function recovery and the adaptability level to the therapist rehabilitation exercises [56,75].

## 6. Conclusions

In this paper, model-based and fuzzy-logic mid-level controllers in hierarchical control of a 1-DoF active shoulder exoskeleton robot for a 2-DoF upper-limb have been simulated. In designing two mid-level controllers, the distinctive effects of the human’s dynamic model, robot’s dynamic model, and the required wrench of the environment (the difference between the estimated torque in the presence of external load and the human dynamic model for free motion) are considered. The controller gains and variables have been optimized for an AAN wearable robot during a task of free motion and lifting in the sagittal plane. The CNS has been simulated with NMPC, and the three mid-level controllers for three phases of (I) initial, (II) short-term, (III) long-term experience of wearing a powered robot have been assessed. The results have shown that model-based and fuzzy-logic controllers could outperform the proportional controller for AAN goals. Based on the results, we propose to use less desired strength in the initial usage of the wearable robots. This hierarchical controller can be used in wearable exoskeletons and rehabilitation robots. The future study trend can focus on implementing and comparing other controllers.

## Figures and Tables

**Table 1:** Fuzzy-logic rule of three functions of the mid-level controller.

#	Condition								Statement			
1	If	$q_{f}$	is	Large Negative (LN)					, then	${\overset{´}{Q}}_{r}$ or ${\overset{´}{Q}}_{h}$	is	Zero (Z)
2				Medium Negative (MN)								Medium (M)
3				Zero (Z)								Large (L)
4				Medium Positive (MP)								Medium (M)
5				Large Positive (LP)								Zero (Z)
6	If	${\overset{´}{\tau }}_{\delta }$	is	Negative (N)	&	$q_{d}-q_{f}$	is	Negative (N)	, then	${\overset{´}{Q}}_{\gamma }$	is	Large Negative (LN)
7				Negative (N)				Zero (Z)				Medium Negative (MN)
8				Negative (N)				Positive (P)				Zero (Z)
9				Zero (Z)				Negative (N)				Small Negative (SN)
10				Zero (Z)				Zero (Z)				Zero (Z)
11				Zero (Z)				Positive (P)				Small Positive (SP)
12				Positive (P)				Negative (N)				Zero (Z)
13				Positive (P)				Zero (Z)				Medium Positive (MP)
14				Positive (P)				Positive (P)				Large Positive (LP)

**Table 2:** The cost function weights and internal model are used for three simulated conditions of (I) initial, (II) short-term, and (III) long-term experience. The 1st and 2nd scenarios are implemented with full and variable strength of the mid-level controllers, respectively (Section 5.1 and Section 5.2).

Experiences		Initial	Short-Term		Long-Term
			1st Scenario	2nd Scenario	
Weights	$\omega_{1}^{T}$ (angle)	$\left[39,183,39,183\right]$	$\left[39,183,39,183\right]$		$\left[40,000,40,000\right]$
	$\omega_{2}^{T}$ (angular velocity)	$\left[1234,1234\right]$	$\left[1234,1234\right]$		$\left[1000,1000\right]$
	$\omega_{3}^{T}$ (torque)	1120,170	1120,170		1200,1200
	$\omega_{4}^{T}$ (torque derivative)	$\left[1360,1360\right]$	$\left[2720,2720\right]$	$\left[2040,2040\right]$	$\left[2000,2000\right]$
IM	human limb dynamic	Yes	Yes		Yes
	known robot’s assistive torque	No	No		Yes

## Data Availability

Not applicable.
